# Review of Reported Adverse Events Associated With Prescription Hearing Aids in the Manufacturer and User Facility Device Experience (MAUDE) Database

**DOI:** 10.7759/cureus.76737

**Published:** 2025-01-01

**Authors:** Quan Lu, Mustafa Husein, Anita Jeyakumar

**Affiliations:** 1 Otolaryngology, Northeast Ohio Medical University, Rootstown, USA; 2 Otolaryngology, HEARS, LLC, Akron, USA

**Keywords:** adverse events, hearing aid, maude database, prescription, side effects

## Abstract

Objective

To examine the adverse events reported in the U.S. Food and Drug Administration's (U.S. FDA’s) Manufacturer and User Facility Device Experience (MAUDE) database for prescription hearing aids (HA).

Methods

A retrospective study was performed from January 2014 to September 2024 using the U.S. FDA’s MAUDE database. Medical device reports (MDRs) were identified using product codes (ESD, OSM, and QDD) and keywords (“Hearing Aid, Air-Conduction, Prescription; Hearing Aid, Air-Conduction with Wireless Technology, Prescription; and Self-fitting, Air-Conduction Hearing Aid, Prescription”). Exclusion criteria included reports unrelated to the usage of the device or those providing insufficient data. The incidence of adverse events was estimated using the Hearing Industries Association’s market data over the last 10 years.

Results

A total of 586 medical device reports (MDRs) were identified, and 504 met the inclusion criteria. There were 4 (0.8%) MDRs identified in 2014, 7 (1.4%) in 2015, 7 (1.4%) in 2016, 11 (2.2%) in 2017, 7 (1.4%) in 2018, 27 (5.4%) in 2019, 50 (9.9%) in 2020, 24 (4.8%) in 2021, 119 (23.6%) in 2022, 174 (34.5%) in 2023, and 74 (14.7%) in 2024, up to the third quarter. Of the 504 MDRs, 829 total events were identified. The mean number of events per MDR was calculated as 1.9. Four hundred and ninety-four (59.6%) of the 829 events were related to device malfunction, with 180 (21.7%) attributed to charger-related issues and 137 (16.5%) to poor construction/device falling apart, resulting in foreign bodies in the ear for 96 (70.1%) of those 137 cases. A total of 303 (36.6%) events were related to adverse medical effects, 19 (2.3%) were related to customer service issues, and 11 (1.3%) were related to failure to provide hearing benefits. The incidence rate of adverse events over the last decade was calculated as 0.00114%.

Conclusions

There is a low reported incidence of adverse events associated with prescription hearing aids. Charger issues, possible poor construction and device failing apart, resulting in foreign bodies in the ear, and medical complications, were commonly reported as adverse events. The data can increase providers' awareness of common complaints, enhance targeted monitoring, and advise and manage expectations.

## Introduction

The National Institute on Deafness and Other Communication Disorders (NIDCD) estimated that 28.8 million adults in the U.S. could benefit from hearing aids, yet only between 16% and 30% of these individuals use them [1,2]. As such, approximately 23 million older individuals with hearing loss are not utilizing and receiving the benefits of hearing aid usage [3]. Multiple reasons have been suggested for these data, including cost, comfort, poor benefit or sound quality, adverse effects, device suitability, and inadequate understanding of how to use the hearing aid [1]. Counseling and proper fitting play a vital role in the successful use of hearing aids. Depending on the patient's needs, various hearing aid options must be considered, including size, style, placement, and circuitry [4]. Each patient will have individual factors to consider, including the size and shape of the ear canal, the extent of hearing loss and power needed for correction, and any features required to address specific hearing challenges. Hearing aids can be obtained over the counter or through a prescription/fitted by an audiologist. Over-the-counter (OTC) hearing aids are more accessible, less expensive, and are Food and Drug Administration (FDA)-approved for mild to moderate hearing loss in individuals over 18 years of age [5]. However, OTC hearing aids offer limited customization. Prescription hearing aids are highly customizable, include professional support, and are better suited for individuals of any age with more complex hearing needs or those requiring advanced technology [6,7].

The U.S. FDA’s Manufacturer and User Facility Device Experience (MAUDE) database compiles medical device reports (MDRs) from mandatory and voluntary sources, including manufacturers, healthcare providers, and patients over the past decade for post-market surveillance. Our previous study used the FDA’s MAUDE database and similar methodology to offer a preliminary report of the adverse events commonly associated with OTC hearing aids [8]. No study to date has analyzed the MDRs specifically involving prescription hearing aids in the FDA’s MAUDE database. Our study aims to identify and describe the adverse events associated with prescription or audiologist-fitted hearing aids over the past decade. By reviewing this data, we strive to improve patient counseling by increasing awareness of the most commonly reported adverse events.

Some of the article's data was submitted for poster presentation at the Asian Pacific American Medical Student Association 2025 National Conference on March 08, 2025.

## Materials and methods

The Northeast Ohio Medical University (NEOMED) institutional review board exempted the current study (Protocol Number 24-013). Data from the FDA’s MAUDE database are de-identified and publicly available. Two independent investigators queried the MAUDE database for all medical device reports (MDRs) related to prescription hearing aids between January 2014 and September 2024 using product codes (ESD, OSM, and QDD) and keywords (“Hearing Aid, Air-Conduction, Prescription; Hearing Aid, Air-Conduction with Wireless Technology, Prescription; and Self-fitting, Air-Conduction Hearing Aid, Prescription”). Duplicated reports were removed, and the remaining MDRs were assessed for suitability for inclusion in the review. Exclusion criteria included reports unrelated to prescription hearing aids and device usage and those providing insufficient information. Information abstracted from the reports consisted of the type of event, year of occurrence, and device manufacturer. Results were then categorized into device malfunction, adverse medical events, customer service-related issues, failure to provide hearing benefits, and interaction with other devices. Two reviewers independently evaluated all free-text descriptions of adverse events in the MDRs and classified the reported events into specific subcategories. MDRs with multiple events were individually classified into suitable categories. The mean number of events per MDR was calculated by dividing the total number of events tabulated by the number of MDRs included in the review. Discussions between the two independent investigators, with inputs from the senior author, were used to resolve any discrepancies that had been identified. Descriptive data analysis, including frequency and percent, and graphical representations were performed using Microsoft Excel (Microsoft Corp., Redmond, WA, US). The total number of prescription hearing aid units sold was estimated based on the data from the Hearing Industries Association on units sold per year between 2014 and 2023, as reported in The Hearing Review and Hearing Tracker [9-16]. For 2024, up to the third quarter, the number of units sold was estimated based on the 2023 number and the compound annual growth rate for the U.S. hearing aid market from the Grand View Research Market Report [17]. The incidence rate was calculated by dividing the number of medical device reports by the total number of prescription hearing aid units sold over the last 10 years and presented as a rate per 100,000 units and as a percentage.

## Results

The MAUDE database searches returned an initial 586 MDRs. Forty-one duplicated reports were removed, 30 were excluded due to insufficient information or unrelated to the prescription hearing aids usage, and 11 were excluded due to incorrect categorization as prescription hearing aids, resulting in 504 MDRs for final review. Most of the reports were from the manufacturers GN Group with 383 (76.0%), WS Audiology with 47 (9.3%), and Sonova with 44 (8.7%). Starkey and Eargo had 10 (2.0%) and five (1.0%) reports, respectively. Siemens and Oticon had four (0.8%) MDRs each, and Integrated Micro-Electronics Inc. had two (0.4%) reports. The remaining manufacturers, Hears.com, Listen Clear, Austar, Natus Medical, and Philips, had one MDR each. There were 4 (0.8%) MDRs identified in 2014, 7 (1.4%) in 2015, 7n (1.4%) in 2016, 11 (2.2%) in 2017, 7 (1.4%) in 2018, 27 (5.4%) in 2019, 50 (9.9%) in 2020, 24 (4.8%) in 2021, 119 (23.6%) in 2022, 174 (34.5%) in 2023, and 74 (14.7%) in 2024, up to the third quarter.

**Figure 1:**
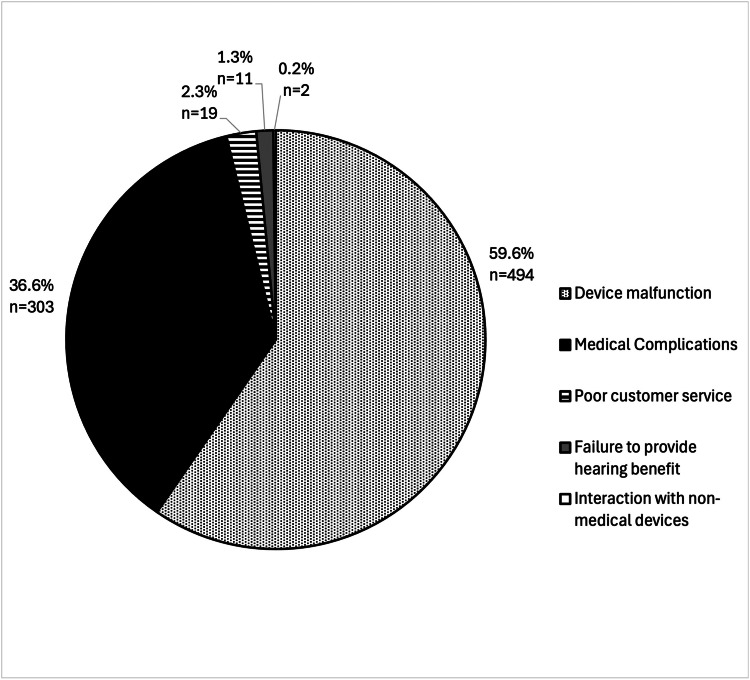
Adverse events of prescription hearing aids as reported in the Manufacturer and User Facility Device Experience (MAUDE) database

Of the 504 MDRs, 829 total events were identified. The mean number of events per MDR was calculated as 1.9. Device malfunction was the most commonly reported adverse event associated with prescription hearing aids at 494 (59.6%) events. Three hundred and three (36.6%) of the 829 reported events were related to medical adverse effects, 19 (2.3%) to customer service-related concerns, 11 (1.3%) to failure to provide hearing benefit, and 2 (0.2%) to interaction with non-medical devices (Figure 1).

The most common device malfunction reported was the hearing aid’s charger overheating, melting, or catching on fire, with 180 incidents (21.7% of the total 829 events or 36.4% of the 494 device malfunction events). One hundred thirty-seven (16.5%) of the 829 events were due to poor construction, device failing apart, or cracking, with 96 (70.1%) of those 137 incidents resulting in a foreign body in the ear (Table 1). Issues with the hearing aids’ dome, receiver, or wax filter/guard comprised most of the 137 poor construction, devices failing apart, or cracking incidents, with 116 (84.7%) reported events. Other less commonly cited device malfunction-related adverse effects included 44 (5.3% of the 829 events) cases of the device emitting noise, 21 (2.5%) cases of charging and battery issues, 20 (2.4%) cases of hearing aid overheating/melting/catching on fire/exploding, 14 (1.7%) cases of feedback, and 18 (2.2%) cases of nonfunctional without explanation (Table 1). The device malfunction’s miscellaneous category included adverse events with low-reported incidence, with the most frequently cited examples of software/programming issues and poor seal/fit/fall from the ear at 13 (1.6%) incidents each (Table 1). Customer service-related complaints were discussed in 19 (2.3%) of the 829 incidents. There were 10 cases of poor communication or difficulty with establishing communication with the manufacturer, 4 reports of dissatisfaction with the product but unable to request a refund, 4 incidents of insufficient customer support, 2 reports each for delayed repair service and not honoring warranty, and 1 case of sending device directly to patient instead of to provider for calibration. Two reports cited possible interaction between the hearing aids and non-medical devices, including the electronic article surveillance system at the store and a battery-operated can opener, which resulted in the hearing aid turning off for 20 minutes and producing a loud ringing noise post-exposure, respectively.

**Table 1:** Adverse events of prescription hearing aids by product codes *: 829 total events, HA: hearing aid, Rx: prescription, Tech: technology

Event Type	HA Rx (ESD)	HA Rx With Wireless Tech. (OSM)	HA Rx, Self-fitting (QDD)	Total Count*	% Total Event
Device malfunction	-	-	-	494	59.6
Poor construction/fell apart/cracked	14	123	0	137	16.5
-> Dome, receiver, wax filter/guard	14	102	0	116	14.0
-> Earmould, shell	0	21	0	21	2.5
-> Foreign body	14	82	0	96	11.6
Charging/battery issue (e.g. Unable to charge, hold charge, internal cell failures, etc.)	3	18	0	21	2.5
Nonfunctional without explanation	11	7	0	18	2.2
Noise emitting from device	3	40	1	44	5.3
Overheated/melted/fire/exploded from HA and/or HA battery	1	19	0	20	2.4
Charger overheated/melted/fire	0	180	0	180	21.7
Feedback	3	11	0	14	1.7
Miscellaneous	-	-	-	60	7.2
Volume adjustment	1	0	0	1	0.1
Receiver wire/tube malfunction	0	3	0	3	0.4
Bluetooth connection/sync issue	1	4	1	6	0.7
Microphone issue	0	2	0	2	0.2
Battery door does not close	0	1	0	1	0.1
Sportlock piece broke off	0	1	0	1	0.1
Excess sound output	0	2	0	2	0.2
Fluctuation in volume	1	3	0	4	0.5
Incorrect calibration	0	2	0	2	0.2
Broken vent	0	1	0	1	0.1
Material protrusion	1	1	0	2	0.2
Poor seal/fit, fall from ear	1	12	0	13	1.6
Product outdated in short time	1	1	0	2	0.2
Software/Programming issue	0	13	0	13	1.6
Background noise	3	4	0	7	0.8
Failure to provide hearing benefit	3	8	0	11	1.3
Customer service concerns	9	10	0	19	2.3
Medical adverse events	-	-	-	303	36.6
Ear infection	6	27	0	33	4.0
Skin concerns (irritation, pruritus, abrasion, redness, edema, inflammation, bleeding, etc)	7	80	0	87	10.5
Sudden reduction/worsening of hearing	5	10	0	15	1.8
Exacerbation of pre-existing condition	0	6	0	6	0.7
Allergic reaction	3	35	0	38	4.6
Dizziness/vertigo	1	6	0	7	0.8
Tinnitus	1	10	0	11	1.3
Neurological symptoms (headache, trigeminal neuralgia, neuritis, CNX stimulation)	1	5	0	6	0.7
Discomfort/pain	10	58	1	69	8.3
Burn (overheating, battery leaking)	2	14	0	16	1.9
Tympanic membrane perforation/injury	1	6	1	8	1.0
Electric shock	1	2	0	3	0.4
Sepsis	0	1	0	1	0.1
Epistaxis	0	1	0	1	0.1
Wax impaction	0	2	0	2	0.2
Interaction with non-medical devices	2	0	0	2	0.2

The most commonly reported medical adverse event for prescription hearing aids was skin complaints, such as irritation, pruritus, abrasion, erythema, edema, inflammation, and bleeding, with 87 (10.5%) incidents out of the 829 total events (Table 1). Discomfort or pain, most often secondary to other adverse events, was reported 69 (8.3%) times. Thirty-eight (4.6%) incidents were attributed to allergic reactions, 33 (4.0%) to ear infections, 16 (1.9%) to burns from overheating or battery leaking, and 15 (1.8%) to sudden reduction/worsening of hearing (Table 1). Other less common medical adverse events identified included tinnitus, tympanic membrane injury, dizziness/vertigo, other neurological symptoms (headache, trigeminal neuralgia, neuritis, cranial nerve X stimulation), electric shock, wax impaction, epistaxis, and sepsis (Table 1).

Per the Hearing Industries Association’s media reports and other publications, the number of hearing aid units dispensed from 2014 to 2023 has been growing from 3.13 million units in 2014 to 5.05 million units in 2023 [9-16]. Based on the estimated compound annual growth rate for the prescription hearing aid of 5.7% from the Grand View Research Report, we estimated that the total number of units sold in 2024, up to the third quarter, will be 4.00 million. As such, the total number of hearing aids dispensed between 2014, and the third quarter of 2024 was an estimated 44.2 million units. The overall incidence rate of adverse events, calculated from the 504 MDRs of adverse events reported in the MAUDE database and 44.2 million total units dispensed, is approximately 1.14 per 100,000 units or 0.00114%. 

## Discussion

Our study provided an overview of the types and frequency of adverse events reported for prescription hearing aids for the past 10 years using the post-market monitoring system of the FDA’s MAUDE database. We identified device malfunction-related complaints followed by medical complications as the most commonly reported adverse events. Charger-related overheating, melting, or fire issues were the most frequently reported adverse events for prescription hearing aids under the device malfunction category in our study, which differed from the literature. For example, recent studies reported that common problems with hearing aids were related to hearing in noisy and windy environments, understanding certain voices, sounds too soft or loud, poor sound quality, feedback noise, and hearing aids failing out of ear [18,19]. Similarly, the National Institute on Deafness and Other Communication Disorders (NIDCD) advises new users of uncomfortable fit, feedback, background noise, user voice inside their head, and buzzing sound with cell phone usage as possible side effects [20]. It was unclear whether the devices, the chargers, or user errors were definitively linked to the reported issues. However, to avoid the charging-related problems, the FDA advises hearing aid users to follow the manufacturer’s guidance, use manufacturer-provided accessories, frequently inspect the device, charge the device away from flammable materials and during the day, unplug the device when fully charged, and protect the device from extreme temperatures [21].

Conversely, some literature reported no adverse events associated with prescription hearing aid usage. A randomized clinical trial of 32 audiologist-fitted devices found no adverse events for the initial six weeks and one requiring gain adjustments at the end of eight months [22,23]. Two Cochrane reviews found limited data on adverse effects, with 1 study of 48 participants with mild to moderate hearing loss and 1 with 91 individuals with hearing loss and concurrent tinnitus reporting no pain or noise-induced hearing loss and no adverse events with device usage, respectively [24,25].

The most commonly reported medical adverse events for prescription hearing aids identified were skin concerns, discomfort/pain, ear infection, burn from overheating device/charger and battery leaking, and sudden reduction in hearing. Similarly, the study by Bennett et al. found that discomfort or ear itching was reported as a concern for 46.8% of the participants [18]. The cross-sectional survey by Machaiah et al. reported tinnitus, itching and/or rashes, and wax accumulation as common adverse effects [19]. Moreover, user guides from the three major manufacturers, GN Group, WS Audiology, and Sonova, listed dizziness, nausea, tinnitus, perceived worsening of hearing, skin reactions, foreign objects in the ear, ear pain, moisture, and cerumen accumulation as possible side effects of hearing aid usage [26-28]. The FDA also warns of adverse events, including skin irritation, injury from the device, burns from overheated battery, foreign objects in the ear, and sudden worsening of hearing [5]. In addition to these medical adverse effects, less described events with hearing aid usage included psychological concerns and social impacts; examples included missing out on conversations, fear of losing/destroying devices and device malfunction, financial stress, people speaking louder, avoiding interaction, and mistreatment of hearing aid users [19].

Our previous study using the FDA’s MAUDE database found that the most commonly reported adverse events for OTC hearing aids were device malfunction resulting in foreign bodies, followed by customer-service-related complaints [8]. There were only 3 (10.0%) reported incidents of medical adverse events, including crusty ear lesions, pruritic ears, and worsening of migraine [8]. The distribution of reported adverse events for prescription hearing aids differed from OTC hearing aids, where device malfunction in the form of charger-related concerns and medical side effects were the most commonly cited events. The FDA officially approved OTC hearing aids for adults in 2022 [7], and perhaps the data is limited due to the decreased period of availability of OTC hearing aids on the market.

Recent effectiveness studies found that self-fitting OTC hearing aids were comparable to audiologist-fitted hearing aids for people with mild to moderate hearing loss, with one participant in the OTC hearing aid group developing a middle ear infection but no adverse events were observed for the prescription group [22,23]. Another study found that the risk of noise-induced hearing loss from overamplification was comparable between prescription and OTC hearing aids [29]. Customer service-related complaints were the second most commonly reported events for OTC hearing aids in the MAUDE database [8], whereas for prescription hearing aids, it was a distant third of commonly reported events. This difference could be attributed to the frequency of follow-ups with users by hearing healthcare providers, especially during the initial months of acclimatization. A recent survey found that hearing aid users required at least one month of heightened attention from the hearing healthcare provider for fine-tuning and reprogramming after startup [30]. In addition, prescription hearing aids have been available on the market longer than OTC hearing aids. As such, the more robust customer support networks could contribute to the differences in adverse events noted. Potential drawbacks of requiring interaction with hearing healthcare providers before purchasing a hearing aid could be the time and financial barriers despite the benefits of ensuring appropriate hearing aid eligibility, fit, programming, and access to support when issues arise. However, we determined that similar to OTC hearing aids, prescription hearing aids also had a low reported incidence of adverse events.

We utilized data from the Hearing Industries Association (HIA), as reported in news releases and online publications, as estimates to determine the number of hearing aid units sold over the last decade. However, the actual number of hearing aids sold and, thus, the estimated incidence of adverse events between 2014 and the third quarter of 2024 remains unclear. The HIA collects data from its members, including manufacturers, suppliers, distributors, and hearing healthcare providers, which could result in underestimating the total units sold by non-member parties. Other considerations when interpreting the incidence of adverse events include under- or over-reporting, inaccurate reporting, and incomplete reporting of adverse events to the FDA database, as consumers, healthcare providers, and manufacturers can all contribute to the MAUDE database. Similarly, the increasing number of adverse event reports over time, as identified by our study, should also be interpreted with caution. Contributing factors to the observed trend could be the increase in patients’ and providers’ awareness of post-market surveillance and willingness to report to the FDA, the number of hearing aid units dispensed over time, and concurrent social and economic events such as the COVID-19 pandemic in 2020 and 2021. Additional research is required to elucidate the trend of adverse events for prescription hearing aids over time. However, adverse effects remain rare incidents for users of prescription hearing aids.

We found two reports of possible prescription hearing aids interacting with non-medical devices. However, it remained unclear from the MDRs whether these events can be attributed to the devices themselves or extraneous factors, given only two isolated incidents during our search and the lack of similar reports in the literature.

The study's limitations included incomplete and insufficient data from the MAUDE database. Since there is no standardization across reports, there is potential for response bias, incomplete information, and lack of consistency. Healthcare professionals, manufacturers, and consumers may report information differently and at different rates. These reports can be difficult to verify, making it challenging to confirm their accuracy. Another limitation of this study is the potential for underreporting, as some reports are voluntary or exempted. As such, the true incidence and prevalence of adverse events still need to be clarified, as voluntary reports depend on the reporter's awareness of the MAUDE database and willingness to submit a report. Despite these limitations, our study provides insight into the adverse effects and user complaints associated with prescription hearing aids, which can help improve hearing aid education, resource, and service allocation to increase hearing aid usage and inspire further research to monitor the safety profile of prescription hearing aids.

## Conclusions

There is a low reported incidence of adverse events for prescription hearing aids. Hearing aid charger malfunction, potential poor construction resulting in foreign bodies in the ear, and medical complaints were the most commonly reported adverse events for prescription hearing aids. The most common adverse medical events included skin concerns, discomfort/pain, allergic reactions, and ear infections. The increasing number of adverse event reports in the MAUDE database may be attributed to improved patient and provider awareness of post-marketing surveillance and an increased number of hearing aids dispensed over the years. This information can increase providers' understanding of common complaints, enhance targeted monitoring and patient assistance, and aid in patient advising and expectations management.
